# Effectiveness of Disease-Specific mHealth Apps in Patients With Diabetes Mellitus: Scoping Review

**DOI:** 10.2196/23477

**Published:** 2021-02-15

**Authors:** Claudia Eberle, Maxine Löhnert, Stefanie Stichling

**Affiliations:** 1 Medicine with Specialization in Internal Medicine and General Medicine Hochschule Fulda–University of Applied Sciences Fulda Germany

**Keywords:** diabetes mellitus, mobile apps, mHealth apps, medical apps

## Abstract

**Background:**

According to the World Health Organization, the worldwide prevalence of diabetes mellitus (DM) is increasing dramatically and DM comprises a large part of the global burden of disease. At the same time, the ongoing digitalization that is occurring in society today offers novel possibilities to deal with this challenge, such as the creation of mobile health (mHealth) apps. However, while a great variety of DM-specific mHealth apps exist, the evidence in terms of their clinical effectiveness is still limited.

**Objective:**

The objective of this review was to evaluate the clinical effectiveness of mHealth apps in DM management by analyzing health-related outcomes in patients diagnosed with type 1 DM (T1DM), type 2 DM (T2DM), and gestational DM.

**Methods:**

A scoping review was performed. A systematic literature search was conducted in MEDLINE (PubMed), Cochrane Library, EMBASE, CINAHL, and Web of Science Core Collection databases for studies published between January 2008 and October 2020. The studies were categorized by outcomes and type of DM. In addition, we carried out a meta-analysis to determine the impact of DM-specific mHealth apps on the management of glycated hemoglobin (HbA_1c_).

**Results:**

In total, 27 studies comprising 2887 patients were included. We analyzed 19 randomized controlled trials, 1 randomized crossover trial, 1 exploratory study, 1 observational study, and 5 pre-post design studies. Overall, there was a clear improvement in HbA_1c_ values in patients diagnosed with T1DM and T2DM. In addition, positive tendencies toward improved self-care and self-efficacy as a result of mHealth app use were found. The meta-analysis revealed an effect size, compared with usual care, of a mean difference of –0.54% (95% CI –0.8 to –0.28) for T2DM and –0.63% (95% CI –0.93 to –0.32) for T1DM.

**Conclusions:**

DM-specific mHealth apps improved the glycemic control by significantly reducing HbA_1c_ values in patients with T1DM and T2DM patients. In general, mHealth apps effectively enhanced DM management. However, further research in terms of clinical effectiveness needs to be done in greater detail.

## Introduction

In today’s world, digitalization is always advancing and increasingly connecting the real with the virtual world [1]. As that happens, our mutual understanding of what is meant by the term digitalization changes. While at the end of the 20th century, digitalization described the conversion of information from analog to digital storage, more extensive definitions are used today [2,3]. For example, a human-centered definition describes digitalization as a process in which people, as well as their living and working worlds, are transferred to a digital level [4]. Digitalization changes the way we interact with our world and vice versa [2]. Consequently, it is not surprising that digitalization also influences the daily lives of patients and health care providers.

Looking back to the 1970s, with the beginning of telematics and telemedicine, the focus was on bridging the distance between patients and health care professionals (HCPs) [2]. However, with the emergence of the internet in the 1990s, new communication channels opened up and the principal use of information and communication technologies became the decisive criterion for digitalization in medicine. The term “electronic health” (eHealth) was created [2,5]. In 2015, the term “digital health” came up in the course of the development and use of new technologies. Digital health includes the use of information and communication technologies to support people in maintaining their health. This is realized by creating opportunities for monitoring, managing, and improving their state of health with the aim of adapting medical care to the needs of the individual [2]. One application of digital health and eHealth is mobile health (mHealth) technologies. mHealth refers to medical and health-promoting methods that are supported by mobile devices such as smartphones and tablets [2,3,5,6]. A smartphone itself can be used as a device to support health, for example via social networking features [3,7]. However, since the launch of smartphone app stores in 2008, it was only a matter of time until apps became a medium for mHealth solutions [3,8,9].

Because the mHealth app market is very heterogeneous and growing so rapidly, there is currently no general mandated definition of mHealth app [10,11]. However, according to the World Health Organization (WHO), mHealth apps are software programs for smartphones and other devices that aim to influence people’s physical, mental, and social well-being in a positive way [12]. In general, medical apps must be distinguished from mHealth apps [13,14]. On a side note, if an mHealth app is classified as a medical app, national and international laws, such as the Medical Device Regulation of the European Union (EU 2017/745), must be taken into account. This means that the app has to go through an approval process that includes, for example, risk analyses [14,15]. Therefore, mHealth apps—medical apps in particular—offer the possibility to improve general health care issues and, more specifically, issues related to type 1 (T1DM) and type 2 (T2DM) diabetes mellitus [3,16-18].

Diabetes mellitus (DM) affects millions of people worldwide and its prevalence is rising [19,20]. Affecting approximately 462 million people globally, T2DM makes up a significant part of the global burden [19], but the prevalence of T1DM, gestational DM (GDM), and other forms of DM are rising drastically as well [20-22]. Despite the huge improvements in diabetes technologies, such as glucose monitoring systems and insulin pumps, many people with diabetes do not meet glycemic control targets [23] and would benefit from greater flexibility and more individualized diabetes therapy.

This underlines the urgent need to improve diabetes care in addition to HCP visits, such as by supporting digital diabetes self-management [24,25]. mHealth apps offer novel possibilities, and first steps have been taken in this regard by a small but growing part of the DM community [26-29]. In 2015, DM-specific mHealth apps had been installed approximately 6.7 million times. Since then, the number of installations has increased dramatically, with approximately 15 million installations in 2018 [29] and 46.3 million installations in 2019 [30], which represented approximately 11% of patients with DM diagnoses worldwide in 2019 [30]. Of the mHealth apps that were installed, 35.8% focused on T1DM, 47.6% on T2DM, and 32.0% on GDM [29]. DM-specific mHealth apps exist in great variety and include different features [31]. Possible app features include tracking of blood glucose levels or insulin usage; calculation of insulin dosages; monitoring of diet, body weight, or physical activities; or providing education or information [3,29,30,32-38]. However, the available evidence on the effectiveness of DM-specific mHealth apps is limited [39]. Therefore, this paper aims to give an overview of the clinical effectiveness of DM-specific mHealth apps on different health-related outcomes for T1DM, T2DM, and GDM. Clinical effectiveness is defined as a process measured by improvements in the parameters of a morbid condition (eg, lowering blood glucose) and aims to provide optimal care, including evidence-based practice [40]. From a clinical point of view, it is important to know the effect size that results from modifying the communication level by using mHealth apps. In clinical practice, these effects must be added to the therapeutic effects (eg, from insulin). This is also important in order to be able to give evidence-based recommendations.

## Methods

### Data Sources and Search Strategy

In October 2020, we conducted a systematic literature search in MEDLINE via PubMed, Cochrane Library, EMBASE, CINAHL, and Web of Science Core Collection in accordance with the Preferred Reporting Items for Systematic Reviews and Meta-Analysis (PRISMA) strategy [41]. These databases are representative of the entire health-related literature on DM, as they are the five largest databases in this field. The search strategy included the following keywords as Medical Subject Headings or EMBASE Subject Headings terms, as well as title and abstract terms: (“diabetes mellitus”) AND (“smartphone” OR “mobile phone” OR “cell phone” OR “iOS” OR “android”) AND (“mobile applications” OR “app”). The search strategy in PubMed, for example, was as follows: (“diabetes mellitus”[Mesh]) AND (“Smartphone”[Mesh]) OR (“Cell Phone”[Mesh]) OR (“mobile phone”[Title/Abstract]) OR (ios[Title/Abstract]) OR (android[Title/Abstract]) AND (app[Title/Abstract]) OR (“Mobile Applications”[Mesh]).

In addition, we manually searched reference lists and Google Scholar to identify further papers. The search results were filtered in the databases by year (January 2008 to October 2020) and language (German and English). The studies were screened and selected by two independent reviewers.

### Eligibility Criteria

Since this is a scoping review, we have included several study designs and outcomes to summarize the evidence available on the topic. We included primary research studies (randomized controlled trials, exploratory studies, observational studies, and pre- and posttest design studies) and peer-reviewed studies published between January 2008 and October 2020. Because English is the worldwide scientific language and the authors are native German, we have taken German and English literature into account.

Studies reporting on the clinical effectiveness of DM-specific mHealth apps in DM management in patients with T1DM, T2DM, and GDM that specified the features of the apps and their health effects were included.

We looked for reported significant changes (*P<*.05) in health-related oucomes such as glycemic control (eg, glycated hemoglobin [HbA_1c_], and hypo- and hyperglycemia), blood pressure, cholesterol, body weight, self-care, and self-efficacy. Self-care was defined and measured as DM self-management that included items assessing general diet, specific diet, exercise, blood glucose testing, foot care, and smoking using a questionnaire. Self-efficacy is a predisposing factor that be impaired in chronic diseases like DM. Increased self-confidence levels, measured by questionnaires, can set the stage for improved glycemic control [42].

Furthermore, we excluded posters, comments, study protocols, duplicates, and studies focused on DM diagnosis or prevention.

### Data Extraction

We extracted the following information about each study: author, year, study design, intervention and control groups, baseline and follow-up HbA_1c_ values, type of DM, sample size, and main findings related to the outcomes of interest.

### Data Synthesis and Analysis

We synthesized the studies according to outcomes because the clinical perspective focuses on the improvement of individual outcomes through the intervention. In addition, we conducted a meta-analysis to assess the impact of the interventions on the management of HbA_1c_.

HbA_1c_ is the most important and most studied clinical outcome related to technological therapy for DM, including mHealth apps. To determine the change in HbA_1c_, we pooled appropriate studies with intervention groups (using mHealth apps only) and control groups (usual care) and calculated the difference in means, with a 95% confidence interval. We included studies that reported changes in HbA_1c_ as a percentage from baseline to the end of the study for intervention and control groups.

## Results

### Overview

**Figure 1:**
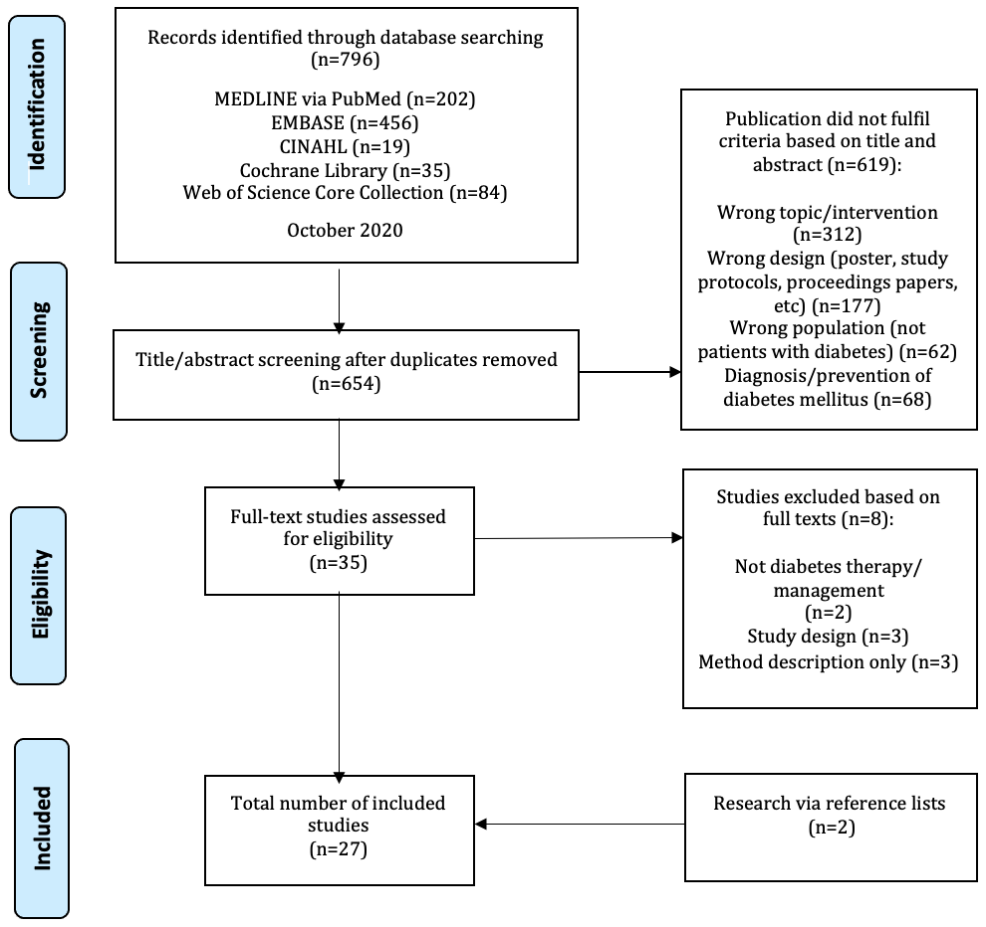
PRISMA (Preferred Reporting Items for Systematic Reviews and Meta-Analyses) flowchart.

The database search in October 2020 in the five relevant databases yielded a total of 796 hits. After removing the duplicates, there were 654 citations. Based on the titles and abstracts, we excluded 619 unsuitable papers. The reasons for exclusion can be found in the PRISMA (Preferred Reporting Items for Systematic Reviews and Meta-Analyses) flowchart (Figure 1). Furthermore, we excluded 8 unsuitable studies based on their full texts. After the additional manual research, which identified 2 papers, there was a total of 27 suitable studies to include in this scoping review. In total, we included 27 papers analyzing 1646 patients in the intervention groups and 1241 in the control groups.

Of the 27 papers, 7 were focused on T1DM (308 patients in the intervention groups and 129 patients in the control groups) [43-49], 12 were focused on T2DM (743 patients in the intervention groups and 645 patients in the control groups) [50-61], and 4 were focused on GDM (339 patients in the intervention groups and 343 patients in the control groups) [62-65]. The remaining 4 papers did not specify the type of DM they looked at or included a mix of DM types (256 patients in the intervention groups and 124 patients in the control groups) [66-69]. Multimedia Appendix 1 gives an overview of the included studies. With regard to the study design, we included 19 randomized controlled trials, 1 randomized crossover trial, 1 exploratory study, 1 observational study, and 5 studies that used a pre-post design (1 of which was controlled). Different diabetes mHealth apps were evaluated in each study. As predicted, the apps had a great variability in their features. Some apps included only one feature, such as digital diaries [47,60], feedback on glucose measurements [51], physical activity promotion [53], data transfer to electronic medical records [61], or educational features [52], while other apps combined multiple features. In the following sections, we present the results of the studies sorted by included outcomes.

### T1DM Studies

#### HbA_1c_

Overall, 264 patients in the intervention groups and 129 patients in the control groups were investigated in the T1DM studies. In 3 of the 7 studies, significant improvements of HbA_1c_ levels within the intervention groups were found (mean difference: –1.1%, *P<*.001 [47]; –0.3%, *P<*.001 [45]; and –0.3%, *P=*.04 [46]), yielding an HbA_1c_ of 7.73% on average. Charpentier et al [48] and Drion et al [43] did not report on significance within groups and Rossi et al [49] did not find significant differences (*P=*.27). Also, 2 studies in which control groups were included reported significant differences between the groups, with better outcomes in the app intervention groups than in the groups receiving usual care (*P<*.001 [47]; –0.67%, *P<*.001 [48]).

#### Hypo- and Hyperglycemia

Foltynski et al [44] found a significant 12% difference in 2-hour postprandial time in range (TIR) in favor of the periods with app support (*P=*.031). However, they did not find significant differences regarding TIR (*P=*.764), time ≤70 mg/dL (*P=*.764), and time ≥180 mg/dL (*P=*.883) [44]. In addition, Tack et al [46] did not find any significant differences in hypoglycemic events (*P=*.21).

#### Fasting Blood Glucose

Fasting blood glucose was reported in 1 study (41 patients [49]), but a significant change was not found (*P=*.09).

#### Self-Care

Kirwan et al [47] used the Summary of Diabetes Self-Care Activities (SDSCA) questionnaire in their study (36 patients in the intervention group, 36 patients in the control group). On the scales for exercise and blood sugar testing, no significant differences were found (*P>*.05). On the scale for diet, there were significant differences within groups (3.42 to 4.62 from baseline to end of study in the intervention group, *P<*.05) but not between groups (1.2 in intervention group versus –0.05 in control group, *P>*.05) [47].

#### Self-Efficacy

Kirwan et al [47] used the Diabetes Empowerment Scale–Short Form (DES–SF) to examine self-efficacy, but no significant differences between the groups were found.

### T2DM Studies

#### HbA_1c_

In total, 743 patients in intervention groups and 645 patients in control groups were investigated in the studies focused on T2DM. Eleven of the studies reported a decrease of HbA_1c_ within the app intervention groups, yielding a mean difference of –0.42% [50-52,54-61], but only 1 study reported a significant change of –1.1% (*P<*.001) [56]. The proportion changes when differences between intervention and control groups were considered. Of 11 studies that included control groups in their study design, 7 studies reported significant differences (mean difference: –0.78%, –1.51 to –0.35) in favor of the app intervention groups [51,53-58], while 3 studies did not find a significant difference between groups [52,59,60] and 1 study did not report on differences between groups [61]. Moreover, Kim et al [61] found a significant decrease of 0.4% (*P<*.001) in HbA_1c_ in their subgroup analysis for participants with a high satisfaction level and no significant decrease in participants with a low satisfaction level.

#### Fasting Blood Glucose

Fasting blood glucose was included in 2 studies (51 patients in the intervention groups and 54 patients in the control groups) [54,57]. Both studies found a significant difference between groups favoring the intervention groups (–28.23 mg/dL, *P<*.001 [54]; –9.6 mg/dL, *P=*.019 [57]).

#### Blood Pressure

None of the 7 studies that reported on blood pressure found significant differences either within or between groups [51-53,55,57,58,61].

#### Cholesterol

Cholesterol levels were reported in 7 studies (407 patients in the intervention groups and 348 patients in the control groups). Six studies looked at total cholesterol [52,53,55,56,58,61], but only 1 study found a significant change within the intervention group (*P=*.01), as well as between the groups (*P=.*009) [56]. High-density lipoprotein (HDL) cholesterol and low-density lipoprotein (LDL) cholesterol were both included in 7 studies [52,53,55-58,61]. Regarding HDL cholesterol, only 1 study found significant differences within groups (*P=*.002 in the intervention group, *P=*.004 in the control group) and between groups, showing greater improvement and lower values in the control group (60.67 mg/dL to 54.33 mg/dL in the intervention group versus 60.07 mg/dL to 52.73 mg/dL in the control group; *P=*.048) [56]. With regard to LDL cholesterol, 1 study reported a significant change within the intervention group (–20.42 mg/dL; *P=*.007) and between the intervention and control groups (*P=*.01) [56].

#### Body Weight

Three studies [52,55,59] observed the body weights of 215 patients in intervention groups and 156 patients in control groups. One study reported a significant difference between the groups (–2.1 kg in the intervention group versus 0.4 kg in the control group; *P=*.021) [51]. While Holmen et al [59] reported a decrease of body weight in the intervention group, they did not report on the significance. Meanwhile, Kim et al [55] did not report significant differences between the intervention and control groups (*P=*.531).

#### Self-Care

Two studies (229 patients in the intervention groups and 224 patients in the control groups) used the SDSCA questionnaire to evaluate self-care [52,54]. Only 1 study reported a significant difference between the groups (*P<*.001) [54]. The scales for diet and exercise were also included in 2 studies [52,54], but only one of the studies showed significant differences between the groups for both outcomes (*P<*.001) [54]. No significant differences were reported for the scales for blood sugar testing (*P=*.509) [52] or smoking (*P=*.729) [54], which were each included in one study.

#### Self-Efficacy

Chomutare et al [50] (7 patients) reported improvements in scores on the DES–SF and Health Education Impact Questionnaire (heiQ [70]), but they did not report on significance. Kusnanto et al [56] (15 patients in the intervention group and 15 patients in the control group) used a diabetes management self-efficacy scale consisting of 15 questions and found significant improvements within and between the groups (within groups: 15.48, *P<*.001 in the intervention group versus 9.6, *P<*.001 in the control group; between groups: *P<*.001).

### GDM Studies

#### HbA_1c_

Two studies [62,64] (167 patients in the intervention groups and 162 in the control groups) investigated the HbA_1c_ levels in patients with GDM. One of the studies [62] found a significant difference between the groups in favor of the app intervention (–1.3% in the intervention group versus –0.6% in the control group; *P<*.001), while the other study found no significant difference [64].

#### Hypo- and Hyperglycemia

Significant differences between groups favoring the app intervention groups were found for off-target fasting glucose measurements (*P<*.001 [62,65]), off-target 1-hour glucose measurements (*P<*.001 [65]), and off-target 2-hour glucose measurements (*P<*.001 [62]).

#### Blood Glucose and Oral Glucose Tolerance Test

Miremberg et al [65] reported a significant difference between the intervention and control groups (*P<*.001), without giving the exact value. Regarding oral glucose tolerance test (OGTT) results, neither Guo et al [62] nor Borgen et al [63] found significant differences in fasting OGTT or 2-hour OGTT.

#### Self-Care

Two studies [62,65] (124 patients in the intervention groups and 120 patients in the control groups) included the outcome of patient compliance, defined as the ratio between actual blood glucose measurements and instructed measurements ×100. Both studies found significant differences between the groups, favoring the app intervention groups (*P<*.001 [62,65]). In addition, Mackillop et al [64] (103 patients in the intervention group and 102 patients in the control group) reported significant differences in the number of blood glucose readings per day, also favoring the app intervention group (*P<*.001).

### Studies With Type of DM not Specified

#### HbA_1c_

Gunawardena et al [66] reported a significant decrease of –0.96% (*P<*.001) in HbA_1c_ level within the app intervention group and a significant difference (*P<*.001) between groups in favor of the intervention group. The study by Yu et al [68] did not show a significant difference in HbA_1c_ between the groups (*P>*.05), but a significant difference was reported regarding the proportion of participants reaching the goal of HbA_1c_ ≤7%, with use of the app as the decisive factor (*P<*.05).

#### Fasting Plasma Glucose

Yu et al [68] reported on fasting plasma glucose (48 patients in the app intervention group and 47 patients in the usual care group), but they found no significant differences between the groups (*P>*.05).

#### Self-Care

Kim et al [69] (90 patients in the intervention group) reported significant improvements through the intervention regarding the total SDSCA score (*P<*.05), as well as on the scales for diet (0.73, *P<*.05), exercise (1.11, *P<*.05), blood sugar testing (1.93, *P<*.05), and smoking (–0.51, *P<*.05). Jeon and Park [67] (38 patients in the intervention group) used the Information-Motivation-Behavioral skills model as a basis to evaluate their app. They found significant improvements in self-care social motivation (*P=*.05) and self-care behaviors (*P=*.02), but they did not find significant differences in self-care information (*P=*.85), self-care personal motivation (*P=*.57), or self-care behavioral skills (*P=*.89) [67].

### Effects on HbA_1c_

Table 1 shows all of the results according to HbA_1c_ values. Effects based on the comparison of HbA_1c_ levels between the intervention and control groups at the study end points were investigated. Findings are presented in Multimedia Appendix 2. The meta-analysis revealed an effect size, compared with usual care, of a mean difference of –0.54% (95% CI –0.8 to –0.28) for T2DM (8 suitable studies) and –0.63% (95% CI –0.93 to –0.32) for T1DM (2 suitable studies) (Multimedia Appendix 3).

**Table 1:** Study results according to glycated hemoglobin (HbA_1c_) values.

			HbA_1c_ (%), mean (SD or 95% CI)			Differences in HbA_1c_ (%): mean (SD or 95% CI), *P* value	
Diabetes type and reference		Study groups	Baseline	Follow up	Within groups		Between groups
**T2DM ^**a**^**							
	[50]	Intervention	6.97 (0.69)	6.79 (0.68)	NR^b^		N/A^c^
	[51]	(A) Intervention; (B) control	(A) 6.86 (1.56); (B) 7.09 (1.51)	NR	(A) –0.40 (–0.67 to –0.14); (B) 0.036 (–0.23 to 0.30)		NR*, P=*.02
	[52]	(A) Intervention; (B) control	(A) 8.1 (1.2); (B) 8.3 (1.6)	(A) 8.0 (1.6); (B) 8.2 (1.4)	NR		–0.08 (–0.37 to 0.2), *P=*.56
	[53]	(A) Intervention; (B) control	(A) 6.2 (0.6); (B) 6.9 (0.7)	(A) 6.2 (0.7); (B) 7.0 (1.0)	NR		–0.9 (–1.5 to –0.2), *P=*.016
	[54]	(A) Intervention; (B) control	(A) 7.10 (1.22); (B) 6.85 (0.93)	(A) 6.84 (0.63); (B) 8.10 (0.10)	(A) NR, *P=*.232; (B) NR, *P<*.001		NR*, P<*.001
	[55]	(A) Intervention; (B) control	(A) 7.7 (0.7); (B) 7.8 (0.7)	NR	(A) –0.4 (0.09); (B) –0.06 (0.1)		0.35 (0.14 to 0.55), *P<*.001
	[56]	(A) Intervention; (B) control	(A) 8.74 (1.34); (B) 8.18 (1.02)	(A) 7.64 (1.29); (B) 7.91 (0.88)	(A) –1.1, *P<*.001; (B) 0.27, *P=*.208		NR, *P=*.005
	[57]	(A) Intervention; (B) control	(A) 7.1 (1.0); (B) 7.0 (0.9)	(A) 6.7 (0.7); (B) 7.1 (1.1)	(A) –0.4; (B) 0.1		NR*, P=*.015
	[58]	(A) Usual care; (B) app only; (C) app + web portal; (D) app + web portal + decision support	(A) 9.2 (1.7); (B) 9.3 (1.8); (C) 9.0 (1.8); (D) 9.9 (2.1)	(A) 8.5 (1.8); (B)7.7 (1.0); (C) 7.9 (1.4); (D) 7.9 (1.7)	(A) –0.7 (–2.3 to –1.0); (B) –1.6 (–2.3 to –1.0); (C) –1.2 (–1.8 to –0.5); (D) –1.9 (–2.3 to –1.5)		A vs D: 1.2 (0.5 to 1.9), *P<*.001; A vs B: NR, *P=*.027; A vs C: NR, *P=*.40
	[59]	(A) Usual care; (B) app; (C) app + HCP^d^ support	(A) 8.4 (7.97 to 8.76); (B) 8.1 (7.72 to 8.53); (C) 8.1 (7.76 to 8.43)	(A) 8.2 (7.77 to 8.61); (B) 7.8 (7.48 to 8.15); (C) 8.0 (7.49 to 8.41)	(A) –0.16 (–0.50 to 0.18); (B) –0.31 (–0.67 to 0.05); (C) –0.15 (–0.58 to 0.29)		A vs. B: –0.22 (–0.75 to 0.32), *P=*.42; A vs C: 0.01 (–0.52 to 0.54), *P=*.097
	[60]	(A) Usual care; (B) app; (C) education program; (D) app + education program	(A) 9.2 (1.6); (B) 9.3 (1.6); (C) 9.4 (1.7); (D) 9.2 (1.4)	NR	(A) –0.7; (B) –0.7; (C) –1.1; (D) –1.1		NR, *P=*.771
	[61]	(A) Intervention; (B) control	(A) 7.7 (0.7); (B) 7.7 (0.5)	(A) 7.5 (0.7); (B) 7.7 (0.7)	(A) NR, *P=*.077; (B) NR, *P=*.973		NR
**T1DM^e^**							
	[43]^f^	(A) Intervention; (B) control	(A) 61 (57 to 65); (B) 62 (57 to 66)	(A) 63 (58 to 67); (B) 63 (57 to 69)	(A) 1 (–1 to 2); (B) 1 (–4 to 6)		–2 (–6 to 5)
	[45]	Intervention	8.1 (7.5 to 9.0)	7.8 (6.9 to 8.3)	NR, *P<*.001		N/A
	[46]	Intervention	7.9	7.6	NR*, P=*.04		N/A
	[47]	(A) Intervention; (B) control	(A) 9.08 (1.18); (B) 8.47 (0.86)	(A) 7.80 (0.75); (B) 8.58 (1.16)	(A) –1.10 (0.74), *P<*.001; (B) 0.07 (0.99), NS^g^		NR, *P<*.001
	[48]	(A) Usual care; (B) app only; (C) app + teleconsultations	(A) 8.91 (0.90); (B) 9.19 (1.14); (C) 9.11 (1.14)	(A) 9.10 (1.16); (B) 8.63 (1.07); (C) 8.41 (1.04)	NR		A vs B: 0.67 (0.35 to 0.99), *P<*.001; A vs C: 0.91 (0.60 to 1.21), *P<*.001; B vs C: *P>*.05
	[49]	Intervention	7.6 (7.3 to 7.9)	NR	–0.33 (–0.77 to 0.11), *P=*.27		N/A
**GDM^h^**							
	[62]	(A) Intervention; (B) control	(A) 6.0 (0.4); (B) 5.9 (0.3)	(A) 4.7 (0.2); (B) 5.3 (0.3)	NR		NR, *P<*.001
	[64]	(A) Intervention; (B) control	(A) 5.42 (0.34); (B) 5.39 (0.35)	NR	(A) 0.02/day; (B) 0.03/day		–0.01 (–0.05 to 0.03), NS

^a^T2DM: type 2 diabetes mellitus.

^b^NR: not reported.

^c^N/A: not applicable.

^d^HCP: health care professional.

^e^T1DM: type 1 diabetes mellitus.

^f^HbA_1c_ values in this study were reported in mmol/mol.

^g^NS: not significant.

^h^GDM: gestational diabetes mellitus.

## Discussion

### Principal Results and Comparison With Prior Work

In general, specific mHealth apps clearly improved glycemic control by effectively reducing HbA_1c_ values in patients with T1DM (mean difference: –0.63%, 95% CI –0.93% to –0.32%) and T2DM (mean difference: –0.54%, 95% CI –0.8% to –0.28%). While no significant improvements in blood pressure or cholesterol were found in patients with T2DM, a few studies showed positive tendencies toward improved self-care and self-efficacy with regard to patients with DM in general.

The studies were diverse with respect to the type of DM, study design, number of participants, and app features. Often, different app features were combined or the app was used in conjunction with web portals, feedback from HCPs, or Bluetooth-enabled devices. Because of that, it was not possible to distinguish a relationship between specific app features and health outcomes.

However, some effects were clearly demonstrated from the use of DM-specific mHealth apps in general. We categorized the outcomes included in the studies into HbA_1c_, hypo- and hyperglycemia, further glycemic control outcomes, blood pressure, cholesterol, body weight, self-care, self-efficacy, and further outcomes.

Nearly all of the studies (22 of 27 studies) included HbA_1c_ level as an outcome, with a total of 2352 patients analyzed. For patients with T1DM, 3 studies reported significant improvements within the intervention groups, with a mean difference of –0.57%, yielding HbA_1c_ levels of 7.73% on average, and 2 studies reported significant differences between groups with a mean difference of –0.73, favoring the intervention groups. Those results are consistent with other reviews. Sun et al [71] reported on 3 studies that showed a significant improvement in HbA_1c_ levels, ranging from –0.50% to –0.58%, in people diagnosed with T1DM. Hou et al [72] reported a significant improvement of –0.49% in HbA_1c_ level but rated the grade of evidence to be low. Moreover, Kitsiou et al [73] investigated the effect of mHealth interventions in general and reported an improvement of –0.3% in HbA_1c_ levels in people with T1DM.

For T2DM, one of the included studies found a significant improvement in HbA_1c_ levels, approximately –1.1%, yielding a mean HbA_1c_ of 7.64% in the intervention group [56], and 7 studies determined a significant difference between intervention and control groups, with a mean difference of –0.78%, favoring the intervention group. Furthermore, Kim et al [61] showed a significant improvement for users who were highly satisfied with the mHealth app. This could be problematic in light of the results of Fu et al [74], who found that patients rated the usability of T2DM-specific apps to be “moderate to catastrophic”. However, Fu et al [74] also reported similar significant improvements in HbA_1c_ values, based on the results of 4 studies, ranging from –1.9% to –0.4% [74]. In addition, they highlighted that people with poor glycemic control (HbA_1c_ >9%) achieved greater reductions and that apps with interactive features (eg, receiving feedback) were especially likely to show highly significant improvements [74]. The importance of receiving feedback, for example from HCPs, was also reported by Hou et al [72]. In their review, they reported that the higher the frequency of HCP feedback was, the greater was the reduction in HbA_1c_ [72]. All in all, they reported a mean difference of –0.57% in HbA_1c_ for patients with T2DM using mHealth apps [72]. In other reviews, such as one by Cui et al [75], a significant mean difference of –0.4% of HbA_1c_ was found between DM-specific mHealth app intervention groups and usual care groups in favor of the intervention groups.

The reported improvements in HbA_1c_ in people with T1DM and T2DM are consistent with the results of the studies that did not specify the type of DM. Of the studies that did not specify the DM type, one study found a significant improvement in HbA_1c_ within the intervention group [66] and the other study found an increase in the proportion of participants with HbA_1c_ <7% [68]. No clear effect on HbA_1c_ could be seen in the studies that focused on patients with GDM because of limited data.

The problem of limited data also applies to the study outcomes of hypo- and hyperglycemia and further glycemic control parameters because the studies included different kinds of outcomes. Thus, no clear conclusions can be drawn from them. Other reviews reported an improvement of glycemic control through mHealth app interventions [71,74,75] but mainly based their conclusions on HbA_1c_ improvements.

The outcomes of blood pressure, cholesterol, and body weight were only included in studies focusing on T2DM. No effect could be determined for blood pressure, total cholesterol, or HDL or LDL cholesterol because the studies predominantly reported nonsignificant differences. With regard to body weight, no effects could be determined either because of inconclusive study results. This is consistent with the review by Cui et al [75], which did not report on the effects of T2DM-specific mHealth apps on blood pressure, cholesterol, or body weight.

Although the data on the outcomes of self-care and self-efficacy were also limited for all types of DM, the studies showed a trend toward improvements in both. Other studies reported improved DM self-management skills as well [71,76]. However, Hoppe et al [77] criticized the lack of inclusion of behavior change techniques in DM-specific mHealth apps.

Other than the effects on health-related outcomes, different aspects of DM-specific mHealth apps should be taken into account for further research and development. For example, Höchsmann et al [53] highlighted that not just the content of an app is important but also the way it conveys the content. They created their app as a game and found significant effects on HbA_1c_ level and steps per day as a result of the intervention [53]. In addition, Boels et al [52] reminded us that the different needs of people with DM—for example, if someone requires insulin or not—need to be considered. Also, the age of the patients appears to matter. Hou et al [78] showed in their subgroup analyses that young people with T2DM are more likely to benefit from apps. Moreover, elderly people diagnosed with DM may have special needs, such as a larger font size because of reduced eyesight, and not all apps are able to meet these needs [79]. This goes hand in hand with the conclusion of Meister et al [2] that living in the digital world demands a kind of digital literacy. But despite the widespread use of smartphones, digital literacy barriers are common in vulnerable populations, which could reduce the effectiveness of diabetes technologies [80]. Moreover, a lack of standards and regulations lead to potential health risks, for example via misinformation through an mHealth app [39]. Certified medical apps are more trustworthy and should therefore be preferred. However, in the field of DM, they are still rare, and additional online libraries of high-quality DM-specific mHealth apps should be taken into account for recommendations [28]. In addition, data safety in mHealth apps is a serious concern, as they deal with sensitive data [28,39,81]. These issues need to be addressed in future studies.

### Limitations of the Study

Although the results of this paper show some possible improvements achieved by using mHealth apps in the treatment of DM, some limitations need to be addressed. A major limitation is the small sample size, especially regarding GDM. Only 4 studies that focused on GDM were included, and they in turn reported predominantly on different outcomes. Thus, no effects of mHealth app use could be determined for patients with GDM. To resolve this issue, we must increase our knowledge of which outcomes are affected by DM-specific mHealth apps and include these outcomes in further studies. In addition, it appears that for patients with GDM, a separate assessment of mHealth app effectiveness is reasonable because outcomes that are important to patients with GDM do not apply in general to patients with T1DM or T2DM, such as different aspects of pregnancy and childbirth. Another limitation of this paper is that the quality of the included studies was not assessed. Therefore, we cannot judge whether an effect was based on poor study quality.

### Conclusions

Overall, this review clearly shows how the use of DM-specific mHealth apps results in improvements in glycemic control by effectively reducing HbA_1c_ levels in patients with T1DM and T2DM. However, a few studies found no significant effects of app use on blood pressure or cholesterol in patients with T2DM. With regard to the other outcomes, only a few suitable studies could be identified. In addition, a handful of studies showed positive tendencies toward improved self-care and self-efficacy as a result of mHealth app use in patients with any type of DM. This suggests a need for further research on the clinical effectiveness of DM-specific mHealth apps.

## Appendix

Multimedia Appendix 1 Overview of included studies.

Multimedia Appendix 2 Results regarding glycated hemoglobin (HbA_1c_) values.

Multimedia Appendix 3 Changes in glycated hemoglobin (HbA_1c_) values (%).

## Abbreviations

DES–SF: Diabetes Empowerment Scale–Short Form
DM: diabetes mellitus
GDM: gestational diabetes mellitus
HCP: health care professional
HDL: high-density lipoprotein
heiQ: Health Education Impact Questionnaire
LDL: low-density lipoprotein
MD: mean difference
mHealth: mobile health
OGTT: oral glucose tolerance test
PRISMA: Preferred Reporting Items for Systematic Reviews and Meta-Analyses
SDSCA: Summary of Diabetes Self-Care Activities
T1DM: type 1 diabetes mellitus
T2DM: type 2 diabetes mellitus
TIR: time in range
