# Supplementary material for: Effectiveness of Disease-Specific mHealth Apps in Patients With Diabetes Mellitus: Scoping Review
Source: JMIR Mhealth Uhealth. 2021 Feb 15;9(2):e23477. doi: 10.2196/23477 (PMC7920757; doi:10.2196/23477)
Supplement: Multimedia Appendix 1 [file mhealth_v9i2e23477_app1.docx]

**Overview of included studies.**

| **Reference** | **Type of Diabetes** | **Study design** | **Participants** | **Included Outcomes** | **Main Features of the app** |
| --- | --- | --- | --- | --- | --- |
| [1] (Chomutare et al. 2013) | T2DM | 1 group pre-post | n=7 | HbA_1c_ (%), Self-efficacy (HeiQ, DES-SF) | Diary, Social features, Connection to glucose monitoring system |
| [2] (Orsama et al. 2013) | T2DM | 2 arm RCT | intervention group: n=24  control group: n=24 | HbA_1c_ (%), body weight, SBP (mmHg), DBP (mmHg) | Feedback |
| [3] (Boels et al. 2019) | T2DM | 2 arm RCT | intervention group: n=115  control group: n=115 | HbA_1c_ (%), Insulin dose (units/kg), total cholesterol (mmol/L), HDL (mmol/L), LDL (mmol/L), SBP (mmHg), DBP (mmHg), self-care (SDSCA: diet, exercise, blood sugar testing) | Education |
| [4] (Höchsmann et al. 2019) | T2DM | 2 arm RCT | intervention group: n=18  control group: n=18 | HbA_1c_ (%), steps/day, total cholesterol (mmol/L), HDL (mmol/L), LDL (mmol/L), SBP (mmHg), DBP (mmHg) | Game for PA promotion |
| [5] (Hooshmandja et al. 2019) | T2DM | 2 arm RCT | intervention group: n=24  control group: n=27 | HbA_1c_ (%), FBS (mg/dL), self-care (SDSCA: total, diet, exercise, smoking) | Education, Diary, Reminder |
| [6] (Kim et al. 2019) | T2DM | 2 arm RCT | intervention group: n=90  control group: n=82 | HbA_1c_ (%), SBP (mmHg), DBP (mmHg), total cholesterol (mmol/L), HDL (mmol/L), LDL (mmol/L), weight (kg), self-care SDSCA, adverse events | Diary, Feedback, Connection with Glucometer, Social Functions |
| [7] (Kusnanto et al. 2019) | T2DM | 2 arm RCT | intervention group: n=15  control group: n=15 | HbA_1c_ (%), self-efficacy (DMSES), total cholesterol (mg/dL), HDL (mg/dL), LDL (mg/dL) | Reminders, Education |
| [8] (Waki et al. 2014) | T2DM | 2 arm RCT | intervention group: n=27  control group: n=27 | HbA_1c_ (%), FBS (mg/dL), HDL (mg/dL), LDL (mg/dL), SBP (mmHg), DBP (mmHg) | Social Functions, Feedback |
| [9] (Quinn et al. 2011) | T2DM | 4 arm cluster RCT | Usual care: n=56  App only: n=23  App+WEB: n=22  App+WEB+ decision support: n=62 | HbA_1c_ (%), total cholesterol (mg/dL), HDL (mg/dL), LDL (mg/dL), SBP (mmHg), DBP (mmHg) | Social Functions, Feedback, Education |
| [10] (Holmen et al. 2014) | T2DM | 3 arm RCT | Usual care: n=50  App: n=51  App+HCP support: n=50 | HbA_1c_ (%), body weight (kg) | Diary, Connection with Glucometer |
| [11] (Forjuoh et al. 2014) | T2DM | 4 arm RCT | Usual care: n=95  App: n=81  Education program: n=101  App+ Education program: n=99 | HbA_1c_ (%) | Diary |
| [12] (Kim et al. 2014) | T2DM | controlled  pre-post | intervention group: n=35  control group: n=35 | HbA_1c_ (%), total cholesterol (mg/dL), HDL (mg/dL), LDL (mg/dL), SBP (mmHg), DBP (mmHg) | Data transfer to EMR |
| [13] (Drion et al. 2015) | T1DM | 2 arm RCT | intervention group: n=31  control group: n=32 | HbA_1c_ (mmol/mol), quality of life | Diary, Reminders |
| [14] (Foltynski et al. 2018) | T1DM | randomized crossover trial | Supported in period 1: n=23  Unsupported in period 1: n=21 | TIR (%), TIR-2h postprandial glycemia (%), T$\leq$70mg/dL (%), T$\geq$180mg/dL (%) | Bolus calculator via speech recognition |
| [15] (Ryan et al. 2017) | T1DM | observational study | n=18 | HbA_1c_ (%) | Diary, Social Functions, bolus calculator |
| [16] (Tack et al. 2018) | T1DM | exploratory study | n=19 | HbA_1c_ (%), Hypoglycemic events/ participant/day, PAID scale, PAID scale-emotional problems | Diary, Social Functions, Reminders, Bolus calculator |
| [17] (Kirwan M. et al. 2015) | T1DM | 2 arm RCT | intervention group: n=36  control group: n=36 | HbA_1c_ (%), self-care (SDSCA: diet, exercise, blood sugar testing), DES-SF | Diary |
| [18] (Charpentier et al. 2011) | T1DM | 3 arm RCT | control group: n=61  App only: n=60  App+ Tele-consultation: n=59 | HbA_1c_ (%) | Bolus calculator, Data transfer to EMR |
| [19] (Rossi et al. 2009) | T1DM | 1 group pre-post | n=41 | HbA_1c_ (%), FBS (mg/dL) | Diary, CHO intake calculator, Social Functions |
| [20] (Guo et al. 2018) | GDM | 2 arm RCT | intervention group: n=64  control group: n=60 | HbA_1c_ (%), OGTT-fasting (mmol/L), OGTT-120min (mmol/L), off-target fasting glucose measurement (%), off-target 2h post-prandial glucose measurement (%), patient compliance (%) | Social Functions, Feedback, Education |
| [21] (Borgen et al. 2019) | GDM | 2 arm RCT | intervention group: n=112  control group: n=121 | OGTT-120min (mmol/L) | Education, Diary, Feedback |
| [22] (Mackillop et al. 2018) | GDM | 2 arm RCT | intervention group: n=103  control group: n=102 | HbA_1c_ (%), blood glucose (mmol/L), blood glucose readings/day | Diary, Social Functions, Education |
| [23] (Miremberg et al. 2018) | GDM | 2 arm RCT | intervention group: n=60  control group: n=60 | patient compliance (%), blood glucose (mg/dL), off-target fasting glucose (%), off-target 1h post-prandial glucose (%) | Diary, Social Functions, Feedback |
| [24] (Gunawardena et al. 2019) | DMx | 2 arm RCT | intervention group: n=35  control group: n=32 | HbA_1c_ (%) | Reminders, Diary, Bolus calculator |
| [25] (Jeon und Park 2019) | DMx | 1 group pre-post | n=38 | self-care social motivation, self-care behavior, self-care information, self-care personal motivation, self-care behavioral skills | Education, Diary, Social Functions, Connection to glucose monitoring system |
| [26] (Yu et al. 2019) | DMx | 4 arm RCT | Control group: n=47  SMBG only: n=45  App only: n=48  SMBG+App: n=45 | HbA_1c_ (%), HbA_1c_ <7% (%), FPG (mmol/mL) | Education, Diary, Social Functions |
| [27] (Kim et al. 2015) | DMx | 1 group pre-post | n=90 | self-care (SDSCA: total, diet, exercise, blood sugar testing, smoking) | Diary, Education |

CHO=carbohydrate, DBP=diastolic blood pressure, DES-SF=Diabetes Empowerment Scale-Short Form, DMSES=diabetes management self-efficacy scale, DMx=no specification of diabetes type, EMR=electronic medical record, FBG=fasting blood glucose, FPG=fasting plasma glucose, GDM=gestational diabetes mellitus, HCP=health care professionals, HDL=high density lipoprotein, HeiQ= Health Education Impact Questionnaire, LDL=low density lipoprotein, OGTT=oral glucose tolerance test, PAID= Problem Areas in Diabetes, SBP=systolic blood pressure, SMBG= self-monitoring of blood glucose, SDSCA= Summary of Diabetes Self-Care Activities, T1DM=type 1 diabetes mellitus, T2DM=type 2 diabetes mellitus, TIR=time in range

References

1. Chomutare T, Tatara N, Årsand E, Hartvigsen G. Designing a diabetes mobile application with social network support. Stud Health Technol Inform 2013;188:58-64. PMID:23823289

2. Orsama A-L, Lähteenmäki J, Harno K, Kulju M, Wintergerst E, Schachner H, Stenger P, Leppänen J, Kaijanranta H, Salaspuro V, Fisher WA. Active assistance technology reduces glycosylated hemoglobin and weight in individuals with type 2 diabetes: results of a theory-based randomized trial. Diabetes Technol Ther 2013;15(8):662-669. PMID:23844570

3. Boels AM, Vos RC, Dijkhorst-Oei L-T, Rutten GEHM. Effectiveness of diabetes self-management education and support via a smartphone application in insulin-treated patients with type 2 diabetes: Results of a randomized controlled trial (TRIGGER study). BMJ Open Diabetes Research and Care 2019;7(1). doi:10.1136/bmjdrc-2019-000981

4. Höchsmann C, Müller O, Ambühl M, Klenk C, Königstein K, Infanger D, Walz SP, Schmidt-Trucksäss A. Novel Smartphone Game Improves Physical Activity Behavior in Type 2 Diabetes. American Journal of Preventive Medicine 2019;57(1):41-50. doi:10.1016/j.amepre.2019.02.017

5. Hooshmandja M, Mohammadi A, Esteghamti A, Aliabadi K, Nili M. Effect of mobile learning (application) on self-care behaviors and blood glucose of type 2 diabetic patients. J Diabetes Metab Disord 2019;18(2):307-313. doi:10.1007/s40200-019-00414-1

6. Kim EK, Kwak SH, Jung HS, Koo BK, Moon MK, Lim S, Jang HC, Park KS, Cho YM. Theeffectofasmartphone-based, patient-centered diabetes care system in patients with type 2 diabetes: A randomized, controlled trial for 24 weeks. Diabetes Care 2019;42(1):3-9. doi:10.2337/dc17-2197

7. Kusnanto, Widyanata KAJ, Suprajitno, Arifin H. DM-calendar app as a diabetes self-management education on adult type 2 diabetes mellitus: a randomized controlled trial. J Diabetes Metab Disord 2019;18(2):557-563. doi:10.1007/s40200-019-00468-1

8. Waki K, Fujita H, Uchimura Y, Omae K, Aramaki E, Kato S, Lee H, Kobayashi H, Kadowaki T, Ohe K. DialBetics: A Novel Smartphone-based Self-management Support System for Type 2 Diabetes Patients. J Diabetes Sci Technol 2014;8(2):209-215. PMID:24876569

9. Quinn CC, Shardell MD, Terrin ML, Barr EA, Ballew SH, Gruber-Baldini AL. Cluster-randomized trial of a mobile phone personalized behavioral intervention for blood glucose control. Diabetes Care 2011;34(9):1934-1942. PMID:21788632

10. Holmen H, Torbjørnsen A, Wahl AK, Jenum AK, Småstuen MC, Årsand E, Ribu L. A Mobile Health Intervention for Self-Management and Lifestyle Change for Persons With Type 2 Diabetes, Part 2: One-Year Results From the Norwegian Randomized Controlled Trial RENEWING HEALTH. JMIR mHealth uHealth 2014;2(4):e57. doi:10.2196/mhealth.3882

11. Forjuoh SN, Bolin JN, Huber Jr JC, Vuong AM, Adepoju OE, Helduser JW, Begaye DS, Robertson A, Moudouni DM, Bonner TJ, McLeroy KR, Ory MG. Behavioral and technological interventions targeting glycemic control in a racially/ethnically diverse population: a randomized controlled trial. BMC Public Health 2014;14(1). doi:10.1186/1471-2458-14-71

12. Kim H-S, Choi W, Baek EK, Kim YA, Yang SJ, Choi IY, Yoon K-H, Cho J-H. Efficacy of the smartphone-based glucose management application stratified by user satisfaction. Diabetes Metab J 2014;38(3):204-210. PMID:25003074

13. Drion I, Pameijer LR, van Dijk PR, Groenier KH, Kleefstra N, Bilo HJG. The Effects of a Mobile Phone Application on Quality of Life in Patients With Type 1 Diabetes Mellitus: A Randomized Controlled Trial. J Diabetes Sci Technol 2015;9(5):1086-1091. PMID:25963412

14. Foltynski P, Ladyzynski P, Pankowska E, Mazurczak K. Efficacy of automatic bolus calculator with automatic speech recognition in patients with type 1 diabetes: A randomized cross-over trial. Journal of Diabetes 2018;10(7):600-608. doi:10.1111/1753-0407.12641

15. Ryan EA, Holland J, Stroulia E, Bazelli B, Babwik SA, Li H, Senior P, Greiner R. Improved A1C Levels in Type 1 Diabetes with Smartphone App Use. Canadian Journal of Diabetes 2017;41(1):33-40. doi:10.1016/j.jcjd.2016.06.001

16. Tack CJ, Lancee GJ, Heeren B, Engelen LJ, Hendriks S, Zimmerman L, Massari D de, Gelder MM, Belt TH. Glucose Control, Disease Burden, and Educational Gaps in People With Type 1 Diabetes: Exploratory Study of an Integrated Mobile Diabetes App. JMIR Diabetes 2018;3(4):e17. doi:10.2196/diabetes.9531

17. Kirwan M., Vandelanotte C., Fenning A., Duncan M.J. Diabetes self-management smartphone application for adults with type 1 diabetes: Randomized controlled trial. Diabetes Technol Ther 2015;17:S56. doi:10.1089/dia.2015.1507

18. Charpentier G, Benhamou P-Y, Dardari D, Clergeot A, Franc S, Schaepelynck-Belicar P, Catargi B, Melki V, Chaillous L, Farret A, Bosson J-L, Penfornis A. The Diabeo software enabling individualized insulin dose adjustments combined with telemedicine support improves HbA1c in poorly controlled type 1 diabetic patients: a 6-month, randomized, open-label, parallel-group, multicenter trial (TeleDiab 1 Study). Diabetes Care 2011;34(3):533-539. PMID:21266648

19. Rossi MCE, Nicolucci A, Pellegrini F, Bruttomesso D, Di Bartolo P, Marelli G, Dal Pos M, Galetta M, Horwitz D, Vespasiani G. Interactive diary for diabetes: A useful and easy-to-use new telemedicine system to support the decision-making process in type 1 diabetes. Diabetes Technol Ther 2009;11(1):19-24. PMID:19132851

20. Guo H, Zhang Y, Li P, Zhou P, Chen L-M, Li S-Y. Evaluating the effects of mobile health intervention on weight management, glycemic control and pregnancy outcomes in patients with gestational diabetes mellitus. J Endocrinol Invest 2019;42(6):709-714. doi:10.1007/s40618-018-0975-0

21. Borgen I, Småstuen MC, van Jacobsen AF, Garnweidner-Holme LM, Fayyad S, Noll J, Lukasse M. Effect of the Pregnant+ smartphone application in women with gestational diabetes mellitus: a randomised controlled trial in Norway. BMJ Open 2019;9(11):e030884. doi:10.1136/bmjopen-2019-030884

22. Mackillop L, Hirst JE, Bartlett KJ, Birks JS, Clifton L, Farmer AJ, Gibson O, Kenworthy Y, Levy JC, Loerup L, Rivero-Arias O, Ming W-K, Velardo C, Tarassenko L. Comparing the Efficacy of a Mobile Phone-Based Blood Glucose Management System With Standard Clinic Care in Women With Gestational Diabetes: Randomized Controlled Trial. JMIR mHealth uHealth 2018;6(3):e71. doi:10.2196/mhealth.9512

23. Miremberg H, Ben-Ari T, Betzer T, Raphaeli H, Gasnier R, Barda G, Bar J, Weiner E. The impact of a daily smartphone-based feedback system among women with gestational diabetes on compliance, glycemic control, satisfaction, and pregnancy outcome: a randomized controlled trial. American Journal of Obstetrics and Gynecology 2018;218(4):453.e1-453.e7. doi:10.1016/j.ajog.2018.01.044

24. Gunawardena KC, Jackson R, Robinett I, Dhaniska L, Jayamanne S, Kalpani S, Muthukuda D. The Influence of the Smart Glucose Manager Mobile Application on Diabetes Management. J Diabetes Sci Technol 2019;13(1):75-81. doi:10.1177/1932296818804522

25. Jeon E, Park H-A. Experiences of Patients With a Diabetes Self-Care App Developed Based on the Information-Motivation-Behavioral Skills Model: Before-and-After Study. JMIR Diabetes 2019;4(2):e11590. doi:10.2196/11590

26. Yu Y, Yan Q, Li H, Li H, Wang L, Wang H, Zhang Y, Xu L, Tang Z, Yan X, Chen Y, He H, Chen J, Feng B. Effects of mobile phone application combined with or without self-monitoring of blood glucose on glycemic control in patients with diabetes: A randomized controlled trial. J Diabetes Investig 2019;10(5):1365-1371. PMID:30815973

27. Kim YJ, Rhee SY, Byun JK, Park SY, Hong S.M., Chin SO, Chon S, Oh S, Woo J, Kim SW, Kim YS. A smartphone application significantly improved diabetes self-care activities with high user satisfaction. Diabetes Metab J 2015;39(3):207-217. doi:10.4093/dmj.2015.39.3.207
