# Supplementary material for: Effectiveness of Disease-Specific mHealth Apps in Patients With Diabetes Mellitus: Scoping Review
Source: JMIR Mhealth Uhealth. 2021 Feb 15;9(2):e23477. doi: 10.2196/23477 (PMC7920757; doi:10.2196/23477)
Supplement: Multimedia Appendix 2 [file mhealth_v9i2e23477_app2.docx]

**Results regarding HbA_1c._**

| **Reference sorted by type of DM** | **Kind of Group** | **Baseline HbA_1c_ (%), mean (SD or 95% CI)** | **Follow up HbA_1c_ (%), mean (SD or 95% CI)** | **Differences within groups HbA_1c_ (%), mean (SD or 95% CI, p)** | **Differences between groups HbA_1c_ (%), mean (SD or 95% CI, p)** |  |
| --- | --- | --- | --- | --- | --- | --- |
| **T2DM** | | | | | | |
| [1] (Chomutare et al. 2013) | Intervention | 6.97 (0.69) | 6.79 (0.68) | NR | N/A |  |
| [2] (Orsama et al. 2013) | Intervention  Control | 6.86 (1.56)  7.09 (1.51) | NR | -0.40 (-0.67 to -0.14)  0.036 (-0.23 to 0.30) | **p=0.022** |  |
| [3] (Boels et al. 2019) | Intervention  Control | 8.1 (1.2)  8.3 (1.6) | 8.0 (1.6)  8.2 (1.4) | NR  NR | -0.08 (-0.37 to 0.2), p=0.557 |  |
| [4] (Höchsmann et al. 2019) | Intervention  Control | 6.2 (0.6)  6.9 (0.7) | 6.2 (0.7)  7.0 (1.0) | NR  NR | -0.9 (-1.5 to -0.2), **p=0.016** |  |
| [5] (Hooshmandja et al. 2019) | Intervention  Control | 7.10 (1.22)  6.85 (0.93) | 6.84 (0.63)  8.10 (0.10) | p=0.232  **p<0.001** | **p<0.001** |  |
| [6] (Kim et al. 2019) | Intervention  Control | 7.7 (0.7)  7.8 (0.7) | NR  NR | -0.4 (0.09)  -0.06 (0.1) | **0.35 (0.14 to 0.55), p<0.001** |  |
| [7] (Kusnanto et al. 2019) | Intervention  Control | 8.74 (1.34)  8.18 (1.02) | 7.64 (1.29)  7.91 (0.88) | **p<0.001**  p=0.208 | **p=0.005** |  |
| [8] (Waki et al. 2014) | Intervention  Control | 7.1 (1.0)  7.0 (0.9) | 6.7 (0.7)  7.1 (1.1) | -0.4  0.1 | **p=0.015** |  |
| [9] (Quinn et al. 2011) | Usual care (A)  App only (B)  App + Web portal (C)  App + Web portal + decision support (D) | 9.2 (1.7)  9.3 (1.8)  9.0 (1.8)  9.9 (2.1) | 8.5 (1.8)  7.7 (1.0)  7.9 (1.4)  7.9 (1.7) | -0.7 (-2.3 to -1.0)  -1.6 (-2.3 to -1.0)  -1.2 (-1.8 to -0.5)  -1.9 (-2.3 to -1.5) | **A vs D: 1.2 (0.5 to 1.9), p<0.001**  **A vs B: p=0.027**  A vs C: p=0.40 |  |
| [10] (Holmen et al. 2014) | Usual care (A)  App (B)  App+ HCP support (C) | 8.4 (7.97 to 8.76)  8.1 (7.72 to 8.53)  8.1 (7.76 to 8.43) | 8.2 (7.77 to 8.61)  7.8 (7.48 to 8.15)  8.0 (7.49 to 8.41) | -0.16 (-0.50 to 0.18)  -0.31 (-0.67 to 0.05)  -0.15 (-0.58 to 0.29) | A vs. B: -0.22 (-0.75 to 0.32), p=0.42  A vs C: 0.01 (-0.52 to 0.54), p=0.097 |  |
| [11] (Forjuoh et al. 2014) | Usual care (A)  App (B)  Education program (C)  App + Education program (D) | 9.2 (1.6)  9.3 (1.6)  9.4 (1.7)  9.2 (1.4) | NR  NR  NR  NR | -0.7  -0.7  -1.1  -1.1 | p=0.771 |  |
| [12] (Kim et al. 2014) | Intervention  Control | 7.7 (0.7)  7.7 (0.5) | 7.5 (0.7)  7.7 (0.7) | p=0.077  p=0.973 | NR |  |
| **T1DM** | | | | | | |
| [13]*(Drion et al. 2015)* | Intervention  Control | 61 (57 to 65)  62 (57 to 66) | 63 (58 to 67)  63 (57 to 69) | 1 (-1 to 2)  1 (-4 to 6) | -2 (-6 to 5) |  |
| [14] (Ryan et al. 2017) | Intervention | 8.1 (7.5 to 9.0) | 7.8 (6.9 to 8.3) | **p<0.001** | N/A |  |
| [15] (Tack et al. 2018) | Intervention | 7.9 | 7.6 | **p=0.04** | N/A |  |
| [16] (Kirwan M. et al. 2015) | Intervention  Control | 9.08 (1.18)  8.47 (0.86) | 7.80 (0.75)  8.58 (1.16) | -1.10 (0.74), p<0.001  0.07 (0.99), ns | **p<0.001** |  |
| [17] (Charpentier et al. 2011) | usual care (A)  App only (B)  App + Tele-consultations (C) | 8.91 (0.90)  9.19 (1.14)  9.11 (1.14) | 9.10 (1.16)  8.63 (1.07)  8.41 (1.04) | NR  NR  NR | A vs B: 0.67 (0.35 to 0.99), **p<0.001**  A vs C: 0.91 (0.60 to 1.21), **p<0.001**  B vs C: p>0.05 |  |
| [18] (Rossi et al. 2009) | Intervention | 7.6 (7.3 to 7.9) | NR | -0.33 (-0.77 to 0.11), p=0.27 | N/A |  |
| **GDM** | | | | | | |
| [19] (Guo et al. 2019) | Intervention  Control | 6.0 (0.4)  5.9 (0.3) | 4.7 (0.2)  5.3 (0.3) | NR  NR | **p<0.001** |  |
| [20] (Mackillop et al. 2018) | Intervention  Control | 5.42 (0.34)  5.39 (0.35) | NR | 0.02%/day  0.03%/day | -0.01 (-0.05 to 0.03), ns |  |
| **DMx** | | | | | | |
| [21] (Gunawardena et al. 2019) | Intervention  Control | 9.25 (1.10)  9.48 (1.22) | 7.2 (0.76)  8.17 (0.85) | between 3 and 6 months:  -**0.96, p<0.001**  -0.45, p=0.45 | p=0.45 (after 3 months)  **p<0.0001** (after 6 months) |  |
| [22] (Yu et al. 2019) | Control group (A)  SMBG only (B)  App only (C)  SMBG + App (D) | 8.7 (1.9)  8.6 (2.0)  8.6 (1.8)  8.3 (1.9) | 7.7 (1.3)  7.7 (1.9)  7.3 (1.3)  7.0 (1.2) | -1.1 (0.4)  -1.1 (0.3)  -1.1 (0.3)  -1.1 (0.3) | p>0.05 |  |

*results in mmol/mol

DMx=no specification of diabetes type, GDM=gestational diabetes mellitus, HCP=health care professionals, N/A=not applicable, NR=not reported, SD=standard deviation, T1DM=type 1 diabetes mellitus, T2DM=type 2 diabetes mellitus

**References**

1. Chomutare T, Tatara N, Årsand E, Hartvigsen G. Designing a diabetes mobile application with social network support. Stud Health Technol Inform 2013;188:58-64. PMID:23823289

2. Orsama A-L, Lähteenmäki J, Harno K, Kulju M, Wintergerst E, Schachner H, Stenger P, Leppänen J, Kaijanranta H, Salaspuro V, Fisher WA. Active assistance technology reduces glycosylated hemoglobin and weight in individuals with type 2 diabetes: results of a theory-based randomized trial. Diabetes Technol Ther 2013;15(8):662-669. PMID:23844570

3. Boels AM, Vos RC, Dijkhorst-Oei L-T, Rutten GEHM. Effectiveness of diabetes self-management education and support via a smartphone application in insulin-treated patients with type 2 diabetes: Results of a randomized controlled trial (TRIGGER study). BMJ Open Diabetes Research and Care 2019;7(1). doi:10.1136/bmjdrc-2019-000981

4. Höchsmann C, Müller O, Ambühl M, Klenk C, Königstein K, Infanger D, Walz SP, Schmidt-Trucksäss A. Novel Smartphone Game Improves Physical Activity Behavior in Type 2 Diabetes. American Journal of Preventive Medicine 2019;57(1):41-50. doi:10.1016/j.amepre.2019.02.017

5. Hooshmandja M, Mohammadi A, Esteghamti A, Aliabadi K, Nili M. Effect of mobile learning (application) on self-care behaviors and blood glucose of type 2 diabetic patients. J Diabetes Metab Disord 2019;18(2):307-313. doi:10.1007/s40200-019-00414-1

6. Kim EK, Kwak SH, Jung HS, Koo BK, Moon MK, Lim S, Jang HC, Park KS, Cho YM. Theeffectofasmartphone-based, patient-centered diabetes care system in patients with type 2 diabetes: A randomized, controlled trial for 24 weeks. Diabetes Care 2019;42(1):3-9. doi:10.2337/dc17-2197

7. Kusnanto, Widyanata KAJ, Suprajitno, Arifin H. DM-calendar app as a diabetes self-management education on adult type 2 diabetes mellitus: a randomized controlled trial. J Diabetes Metab Disord 2019;18(2):557-563. doi:10.1007/s40200-019-00468-1

8. Waki K, Fujita H, Uchimura Y, Omae K, Aramaki E, Kato S, Lee H, Kobayashi H, Kadowaki T, Ohe K. DialBetics: A Novel Smartphone-based Self-management Support System for Type 2 Diabetes Patients. J Diabetes Sci Technol 2014;8(2):209-215. PMID:24876569

9. Quinn CC, Shardell MD, Terrin ML, Barr EA, Ballew SH, Gruber-Baldini AL. Cluster-randomized trial of a mobile phone personalized behavioral intervention for blood glucose control. Diabetes Care 2011;34(9):1934-1942. PMID:21788632

10. Holmen H, Torbjørnsen A, Wahl AK, Jenum AK, Småstuen MC, Årsand E, Ribu L. A Mobile Health Intervention for Self-Management and Lifestyle Change for Persons With Type 2 Diabetes, Part 2: One-Year Results From the Norwegian Randomized Controlled Trial RENEWING HEALTH. JMIR mHealth uHealth 2014;2(4):e57. doi:10.2196/mhealth.3882

11. Forjuoh SN, Bolin JN, Huber Jr JC, Vuong AM, Adepoju OE, Helduser JW, Begaye DS, Robertson A, Moudouni DM, Bonner TJ, McLeroy KR, Ory MG. Behavioral and technological interventions targeting glycemic control in a racially/ethnically diverse population: a randomized controlled trial. BMC Public Health 2014;14(1). doi:10.1186/1471-2458-14-71

12. Kim H-S, Choi W, Baek EK, Kim YA, Yang SJ, Choi IY, Yoon K-H, Cho J-H. Efficacy of the smartphone-based glucose management application stratified by user satisfaction. Diabetes Metab J 2014;38(3):204-210. PMID:25003074

13. Drion I, Pameijer LR, van Dijk PR, Groenier KH, Kleefstra N, Bilo HJG. The Effects of a Mobile Phone Application on Quality of Life in Patients With Type 1 Diabetes Mellitus: A Randomized Controlled Trial. J Diabetes Sci Technol 2015;9(5):1086-1091. PMID:25963412

14. Ryan EA, Holland J, Stroulia E, Bazelli B, Babwik SA, Li H, Senior P, Greiner R. Improved A1C Levels in Type 1 Diabetes with Smartphone App Use. Canadian Journal of Diabetes 2017;41(1):33-40. doi:10.1016/j.jcjd.2016.06.001

15. Tack CJ, Lancee GJ, Heeren B, Engelen LJ, Hendriks S, Zimmerman L, Massari D de, Gelder MM, Belt TH. Glucose Control, Disease Burden, and Educational Gaps in People With Type 1 Diabetes: Exploratory Study of an Integrated Mobile Diabetes App. JMIR Diabetes 2018;3(4):e17. doi:10.2196/diabetes.9531

16. Kirwan M., Vandelanotte C., Fenning A., Duncan M.J. Diabetes self-management smartphone application for adults with type 1 diabetes: Randomized controlled trial. Diabetes Technol Ther 2015;17:S56. doi:10.1089/dia.2015.1507

17. Charpentier G, Benhamou P-Y, Dardari D, Clergeot A, Franc S, Schaepelynck-Belicar P, Catargi B, Melki V, Chaillous L, Farret A, Bosson J-L, Penfornis A. The Diabeo software enabling individualized insulin dose adjustments combined with telemedicine support improves HbA1c in poorly controlled type 1 diabetic patients: a 6-month, randomized, open-label, parallel-group, multicenter trial (TeleDiab 1 Study). Diabetes Care 2011;34(3):533-539. PMID:21266648

18. Rossi MCE, Nicolucci A, Pellegrini F, Bruttomesso D, Di Bartolo P, Marelli G, Dal Pos M, Galetta M, Horwitz D, Vespasiani G. Interactive diary for diabetes: A useful and easy-to-use new telemedicine system to support the decision-making process in type 1 diabetes. Diabetes Technol Ther 2009;11(1):19-24. PMID:19132851

19. Guo H, Zhang Y, Li P, Zhou P, Chen L-M, Li S-Y. Evaluating the effects of mobile health intervention on weight management, glycemic control and pregnancy outcomes in patients with gestational diabetes mellitus. J Endocrinol Invest 2019;42(6):709-714. doi:10.1007/s40618-018-0975-0

20. Mackillop L, Hirst JE, Bartlett KJ, Birks JS, Clifton L, Farmer AJ, Gibson O, Kenworthy Y, Levy JC, Loerup L, Rivero-Arias O, Ming W-K, Velardo C, Tarassenko L. Comparing the Efficacy of a Mobile Phone-Based Blood Glucose Management System With Standard Clinic Care in Women With Gestational Diabetes: Randomized Controlled Trial. JMIR mHealth uHealth 2018;6(3):e71. doi:10.2196/mhealth.9512

21. Gunawardena KC, Jackson R, Robinett I, Dhaniska L, Jayamanne S, Kalpani S, Muthukuda D. The Influence of the Smart Glucose Manager Mobile Application on Diabetes Management. J Diabetes Sci Technol 2019;13(1):75-81. doi:10.1177/1932296818804522

22. Yu Y, Yan Q, Li H, Li H, Wang L, Wang H, Zhang Y, Xu L, Tang Z, Yan X, Chen Y, He H, Chen J, Feng B. Effects of mobile phone application combined with or without self-monitoring of blood glucose on glycemic control in patients with diabetes: A randomized controlled trial. J Diabetes Investig 2019;10(5):1365-1371. PMID:30815973
