# Supplementary material for: Effectiveness of Disease-Specific mHealth Apps in Patients With Diabetes Mellitus: Scoping Review
Source: JMIR Mhealth Uhealth. 2021 Feb 15;9(2):e23477. doi: 10.2196/23477 (PMC7920757; doi:10.2196/23477)
Supplement: Multimedia Appendix 3 [file mhealth_v9i2e23477_app3.docx]

**Changes in HbA_1c_ values (%)**

| **Study** | **Intervention group, HbA_1c_ at endpoint** | **Control group, HbA_1c_ at endpoint** | **Mean difference** |
| --- | --- | --- | --- |
| **Type 2 Diabetes** | | | |
| Boels et al. 2019 | **8** | **8,2** | -0,2 |
| Höchsmann et al. 2019 | **6,2** | **7** | -0,8 |
| Hooshmandja et al. 2019 | **6,84** | **8,1** | -1,26 |
| Kusnanto et al. 2019 | **7,64** | **7,91** | -0,27 |
| Waki et al. 2014 | **6,7** | **7,1** | -0,4 |
| Quinn et al. 2011 | **7,7** | **8,5** | -0,8 |
| Holmen et al. 2014 | **7,8** | **8,2** | -0,4 |
| Kim et al. 2014 | **7,5** | **7,7** | -0,2 |
| **MD** |  |  | **-0,54125** |
| **SD** |  |  | **0,377375807** |
| **Upper limit** |  |  | **-0,279741907** |
| **Lower limit** |  |  | **-0,802758093** |
| **Type 1 Diabetes** | | | |
| Kirwan et al. 2015 | **7,8** | **8,58** | -0,78 |
| Charpentier et al. 2011 | **8,63** | **9,1** | -0,47 |
| **MD** |  |  | **-0,625** |
| **SD** |  |  | **0,219203102** |
| **Upper limit** |  |  | **-0,3212** |
| **Lower limit** |  |  | **-0,9288** |
